# Prediction of 30-Day All-Cause Hospital Readmissions Using Limited Structured Electronic Health Record Data: Retrospective Comparative Study

**DOI:** 10.2196/83918

**Published:** 2026-05-22

**Authors:** Ritam Ghosh, Dariush Khezrimotlagh, Sara Imanpour, Ilya Shvartsman

**Affiliations:** 1 School of Science, Engineering, and Technology, Pennsylvania State University Harrisburg, Olmsted Building W255, 777 West Harrisburg Pike, Middletown, PA, United States, 1 7179486179; 2 School of Business Administration, Pennsylvania State University, Harrisburg, PA, United States

**Keywords:** 30-day readmission, clinical decision support systems, current procedural terminology, digital health, early prediction, electronic health records, health risk assessment, *International Classification of Diseases*, machine learning, retrospective studies

## Abstract

**Background:**

Unplanned hospital readmissions represent a critical operational and financial challenge for health care systems in the United States, with 3.8 million 30-day all-cause readmissions in 2018 at an average cost of US $15,200 each, totaling US $58 billion in costs. Many published prediction models rely on comprehensive information (eg, full billing abstractions, discharge summaries, laboratory tests, and vitals) that becomes available only late in the encounter, limiting usefulness for real-time, in-hospital intervention. This creates a timeliness-accuracy trade-off: models that are most accurate retrospectively may arrive too late to act upon.

**Objective:**

This study tests whether a clinically meaningful predictive signal for 30-day all-cause readmission is present within a limited set of structured clinical codes recorded during the patient’s hospital stay. This approach evaluates whether predictive signals are retained when using a restricted set of structured clinical codes.

**Methods:**

We conducted a retrospective comparative modeling study using a large, deidentified electronic health record dataset of 50,000 inpatient encounters from the 2019 New York State Emergency Department Database. Two feature sets were constructed: (1) a limited set consisting of the first 5 *ICD-10* (*International Classification of Diseases, 10th Revision*) diagnosis codes, the first 5 Current Procedural Terminology (CPT) codes, and Charlson Comorbidity Index (CCI; 11 input features); and (2) a rich set using all available *ICD-10* and CPT codes plus CCI (up to 135 input features). We trained 4 models: random forest, CatBoost, multilayer perceptron, and DistilBERT (a distilled Bidirectional Encoder Representations from Transformers [BERT] model; structured codes mapped to text and tokenized with DistilBERT-base-uncased). Evaluation used an untouched hold-out set of 10,000 encounters, preserving the natural 21.1% readmission prevalence. Primary metrics were area under the receiver operating characteristic curve (AUROC), *F*_1_-score, and accuracy. To address class imbalance, the training split only was balanced via undersampling of the majority class and bootstrap oversampling of the minority class; validation/test distributions were left unchanged.

**Results:**

Models trained on the limited feature set achieved AUROC values ranging from 0.5369 to 0.5596 and *F*_1_-scores from 0.2555 to 0.3434. Across 3 of 4 architectures, models trained on the limited feature set matched or exceeded the discrimination of their rich counterparts. The best model (random forest, limited) achieved an area under the curve AUROC 0.5596 (95% CI 0.5440‐0.5739) compared to the best performing rich model (DistilBERT) at 0.5703 (95% CI 0.5565‐0.5842), an absolute difference of 0.0107. The highest *F*_1_-score (0.3434) was achieved by DistilBERT on the limited feature set. Differences across architectures were small in absolute terms, with threshold-dependent metrics (eg, *F*_1_-score) being comparable.

**Conclusions:**

The findings suggest that models using a limited set of structured clinical codes can achieve performance comparable to those using more comprehensive coding information.

## Introduction

In the United States health care system, few challenges carry the same clinical and financial weight as unplanned 30-day all-cause hospital readmissions. These events are not only indicators of potential gaps in care quality but also represent a significant source of patient harm due to inferior quality of patient care and culminate in an estimated cost of US $35.7 billion to the Medicare program in 2018 alone [1]. The implementation of federal policies like the Hospital Readmissions Reduction Program (HRRP), which imposes substantial financial penalties on hospitals with high readmission rates, has transformed this issue from a quality improvement goal into an urgent financial and operational mandate for health care administrators [2,3].

The scale of the problem is staggering, with an estimated 3.8 million adults experiencing a readmission within 30 days of discharge each year [4]. Each of these events carries an average cost of approximately US $15,200 and over US $58 billion for the US health care system as a whole, a figure corroborated by a recent meta-analysis from Ghabowen et al [5] that found a mean cost of US $16,037 [6].

This immense financial pressure has been codified into federal policy through the Patient Protection and Affordable Care Act’s HRRP [7]. Enacted in 2012 [2], the HRRP fundamentally altered the reimbursement landscape by directly linking a hospital’s financial health to its readmission performance. The program imposes significant penalties on hospitals with higher-than-expected readmission rates, which can amount to a loss of up to 3% of their total base Medicare inpatient payments, a penalty applied across the board, not just to the specific cases that resulted in readmission [3,8]. The impact of this policy has been widespread and enduring. In its inaugural year, the HRRP penalized 2217 hospitals. A decade later, in fiscal year 2023, the program penalized 2273 hospitals, nearly three-quarters of those evaluated, for a total of US $320 million [8].

For the purposes of policy and research, the most widely accepted definition is provided by the Centers for Medicare & Medicaid Services (CMS). A 30-day all-cause readmission is defined as an unplanned admission for any reason to a short-stay, acute-care hospital within 30 days of discharge from an initial hospitalization, and throughout the manuscript, we use the term “30-day all-cause readmission” to refer exclusively to this definition [9]. The “all-cause” nature means that a patient discharged after treatment for heart failure who is subsequently hospitalized for a fall still counts as a readmission, making the predictive task inherently difficult as it requires capturing a state of general vulnerability rather than a specific disease trajectory.

The quest to predict and prevent these costly events has spurred a significant evolution in risk stratification methodologies. Early efforts centered on simple, rule-based scoring systems, the most prominent of which is the length of stay, acuity of the admission, comorbidity of the patient, and emergency department use (LACE) index, first developed by van Walraven et al [10]. This tool calculates a risk score based on 4 easily accessible variables: length of stay, acuity of the admission, comorbidities (via Charlson Comorbidity Index [CCI]), and the number of emergency department visits in the preceding 6 months [10]. The appeal of LACE is its simplicity and its reliance on routinely collected administrative data, making it easy for hospital staff to calculate. However, its widespread use overlooks significant limitations. While some studies report fair discriminatory power, with c-statistics (a measure of model accuracy, also known as area under the curve [AUROC]) in the range of 0.63 to 0.68 [10], other research suggests it offers little added value over a clinician’s own judgment [11]. A crucial flaw, identified in a retrospective cohort study by Damery and Combes [10], is that even when a high LACE score is predictive, it may only identify a small fraction, approximately 25%, of all patients who are ultimately readmitted [11]. This leaves the vast majority of at-risk patients unflagged, creating a dangerous blind spot for any resource allocation strategy based on the tool. The simplicity of LACE is, therefore, both its greatest strength and its fatal flaw; by reducing the multifaceted drivers of readmission to a simple additive score, it is structurally incapable of capturing the complex, nonlinear interactions that truly define a patient’s risk, creating a clear justification for more sophisticated approaches.

To overcome these limitations, the field has increasingly turned to machine learning (ML) techniques, including logistic regression, support vector machines, and ensemble methods like random forest and gradient boosting, capable of analyzing high-dimensional data to uncover subtle patterns missed by static indices. This shift, however, has led to a complex and often paradoxical body of evidence. While many studies report that ML models outperform traditional methods, systematic reviews reveal that the magnitude of this improvement is often modest and inconsistent. One meta-analysis found that while ML approaches did perform better, the average improvement in AUROC was only 0.03 [12]. In another comprehensive review, Christodoulou et al [12] concluded there was no significant overall performance benefit of ML over logistic regression for clinical prediction models [13]. A recent systematic review by Sharda et al [13] found that specific models like artificial neural networks and random forests were particularly effective, while approaches using natural language processing showed more limited success [14]. Reported AUROCs vary widely, from as high as 0.88 in studies focused on specific, high-risk cohorts with rich data sources to a more modest range of 0.60 to 0.63 in studies examining all-cause readmissions in general populations [14,15]. Several studies have explored electronic health record (EHR)–based or ML approaches for readmission prediction using structured features or simpler representations [16,17].

A central challenge that persists across these approaches is the “timeliness-accuracy trade-off,” an inverse relationship between when a prediction is available and how accurate it can be. Many of the highest-performing models achieve their accuracy by using comprehensive clinical data, such as full billing records and discharge summaries, which are only available after a patient has left the hospital [18-20]. A model that predicts readmission risk with high accuracy after the patient has already left the hospital is of little use for triggering the in-hospital interventions, such as a specialized pharmacist consultation, social worker engagement, or enhanced care transition planning, that could have prevented the readmission in the first place.

This study is designed to test whether a clinically meaningful predictive signal for 30-day-all-cause readmission is concentrated within a limited set of structured clinical codes recorded during the patient’s hospital encounter. To test this hypothesis, we train and evaluate 4 ML architectures: random forest, CatBoost, a multilayer perceptron (MLP) with entity embeddings, and a distilled Bidirectional Encoder Representations from Transformers (DistilBERT) model adapted for structured data, on a large-scale, deidentified dataset of 50,000 patient encounters. Each model is tested under 2 scenarios: a “limited feature set” consisting of the first *5 ICD-10* (*International Classification of Diseases, 10th Revision*) and 5 Current Procedural Terminology (CPT) codes plus the CCI, and a “rich feature set” including all available codes. The primary contribution of this work is the empirical evaluation of predictive performance across restricted and more comprehensive structured coding representations.

## Methods

### Data Source and Study Cohort

The data for this study was sourced from the 2019 New York State Emergency Department Database (SEDD), part of the Healthcare Cost and Utilization Project (HCUP), sponsored by the Agency for Healthcare Research and Quality. The dataset consists of deidentified administrative records derived from hospital billing and discharge data across participating hospitals within the state and does not contain direct personal identifiers. The raw source data consisted of 6,730,523 rows of diagnosis entries linked to hospital encounters rather than a single encounter table. For this study, cohort construction was performed using the VisitLink identifier to define encounters. Eligible encounters were defined as those with a valid 8-digit VisitLink and a nonmissing DaysToEvent value. The raw dataset contained 6,730,523 rows, of which 1,223,985 (18.2%) records lacked a valid VisitLink and 210 (0.003%) records had missing DaysToEvent, resulting in the exclusion of 1,224,195 rows during data preparation. No additional exclusion criteria were applied beyond these eligibility definitions. For diagnosis and procedure code variables, missingness primarily reflects structural absence as fewer recorded codes rather than missing data, and these values were retained without imputation. From this eligible pool, 50,000 encounters were randomly selected to construct the analytic dataset for this initial comparative analysis. Figure S1 in Multimedia Appendix 1 summarizes these preprocessing and sampling steps. Because the source data consist of encounter-linked records rather than a preassembled encounter-level dataset, the diagram focuses on eligibility definition and sampling rather than stepwise exclusion counts. The variables used in the analysis included diagnosis codes (*ICD-10*), procedure codes (CPT), the readmission outcome derived from DaysToEvent, and CCI. No clinical measurements such as laboratory results, vital signs, or clinical notes were included; the models rely solely on structured administrative coding data.

The limited feature set retained only the first *5 ICD-10* and first 5 CPT code positions, whereas the rich feature set retained all available code positions up to the predefined limits. Because explicit timestamps are not available, code position order was used, and only the first 5 *ICD-10* and CPT code positions are included to avoid incorporating information recorded later in the encounter.

The primary outcome for our predictive models was 30-day all-cause emergency department revisit, defined as any subsequent emergency department encounter for the same VisitLink occurring within 30 days of the index encounter, regardless of hospital. No distinction between planned and unplanned revisits was possible based on the available data.

The first, the limited feature set, included only the first 5 CPT codes, the first 5 *ICD-10* codes, and the calculated CCI score for each patient (11 input features). The second, the rich feature set, included all available codes for each encounter, up to 100 CPT codes and 34 *ICD-10* codes, in addition to the CCI (up to 135 input features). The thresholds (5 CPT and 5 *ICD-10* for the limited set; 100 CPT and 34 *ICD-10* for the rich set) were selected based on the observed distribution of code counts in the dataset. The rich feature set was used as a comparator, including all available coding information.

### Model Development and Evaluation

**Figure 1.**
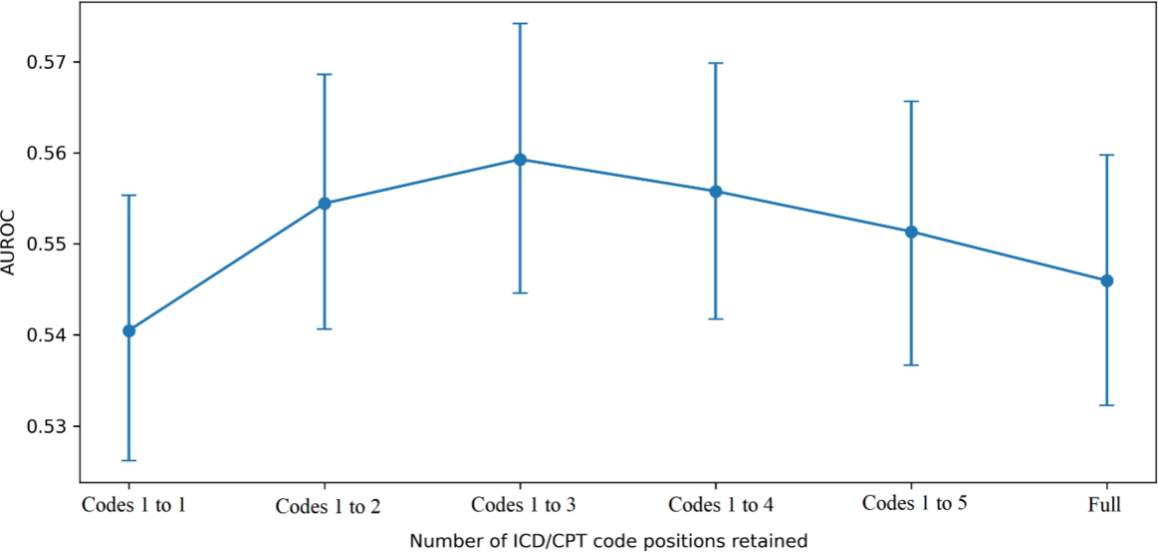
Area under the receiver operating curve (AUROC) as a function of the number of retained *International Classification of Diseases* (*ICD*) and Current Procedural Terminology (CPT) code positions.

The methodology for this study was structured as a rigorous comparative experiment to systematically assess the predictive performance of four distinct ML models when applied to two different feature sets. The entire experimental workflow, from data preprocessing to final model evaluation, was carefully carried out to ensure a fair comparison, as illustrated in the overall workflow diagram in Figure 1.

The process began with several data preprocessing steps to prepare the raw EHR data for modeling. To create a standardized measure of patient comorbidity, each encounter’s diagnosis codes (*ICD-10*) were converted into a CCI score. Specifically, diagnosis fields were coerced to strings with blanks for missing values, and we used a reproducible Python pipeline (the icd package) to apply the established Charlson/Deyo algorithm with Quan’s updated *International Classification of Diseases* mappings (*ICD-10* “charlson10” map). The mapping produced per-encounter indicator flags for Charlson conditions, which were then combined using the conventional Charlson weights; standard hierarchy rules were enforced to avoid double counting. The resulting integer CCI score was merged back to the encounter table by ID in a deterministic, rule-based manner. To ensure consistency for the modeling algorithms, all diagnosis (*ICD-10*) and procedure (CPT) code fields were normalized by converting them to uppercase and stripping any nonalphanumeric characters. Finally, because the number of codes per encounter varied, records were padded with null values to create a fixed-length representation, such that encounters with fewer than the maximum number of codes were supplemented with null placeholders, ensuring uniform input dimensions for all models. No features were dropped due to low variance, missingness, or collinearity; all structured codes in both feature sets were retained.

To ensure an unbiased evaluation, the full dataset of 50,000 encounters was first partitioned into a training set (n=40,000, 80% encounters) and a test set (n=10,000, 20% encounters). This division was performed using a stratified split to maintain the original 21.1% readmission rate in both subsets, ensuring the test set accurately reflected the real-world data distribution.

The test set was then held out and remained untouched until the final evaluation phase. A significant challenge in the training data was the substantial class imbalance, with nonreadmissions outnumbering readmissions by nearly 4 to 1. To address class imbalance, we created a balanced training set using a hybrid of undersampling and bootstrap oversampling implemented in Python (pandas and Hugging Face Datasets). After splitting the data, we converted the training split to a pandas DataFrame and undersampled the majority class (nonreadmissions) to 15,000 instances without replacement. We then bootstrap-oversampled the minority class (readmissions) to 15,000 instances with replacement. The 2 subsets were concatenated and shuffled, yielding a balanced training set of 30,000 records. We preserved the original test (and validation) distributions; no resampling was applied outside the training split to avoid target leakage. This process yielded a perfectly balanced training dataset of 30,000 encounters with a 1:1 class ratio, which was used to train all subsequent models. No class weighting or threshold adjustment was applied. All hyperparameter tuning was performed exclusively on training and validation subsets, with no access to the test set at any stage.

Four distinct ML models were trained on both the limited- and rich-feature sets, each requiring a unique data preparation pipeline to accommodate its architecture. The first model was a random forest classifier, configured with 200 trees and a maximum tree depth of 20, with hyperparameters tuned via grid search with 3-fold cross-validation on the training set. For this model, the categorical *ICD-10* and CPT codes were transformed using ordinal encoding. The second model was CatBoost, a gradient-boosted decision tree algorithm 5 that was trained for 1000 iterations with a learning rate of 0.05, a tree depth of 6. To prevent overfitting, we used early stopping on a stratified validation subset (10% of the training split): training halted if the validation *F*_1_-score failed to improve for 50 consecutive iterations, and the best-iteration weights were restored (ie, the model at the peak validation *F*_1_-score was retained).

The third model was an MLP, a type of neural network. Here, each CPT and *ICD-10* code was first ordinally encoded and then mapped to a low-dimensional vector using an entity embedding layer. The network architecture consisted of 2 hidden dense layers (with 256 and 128 neurons, respectively) using Rectified Linear Unit activation, with BatchNorm and a dropout rate of 0.4 applied for regularization. The model was trained for 15 epochs using the Adam optimizer.

The final and most distinct model was DistilBERT, which required a unique, multistage data transformation pipeline to reframe the structured clinical codes as natural language codes into a narrative text, which is then tokenized for model input.

**Figure 2.**
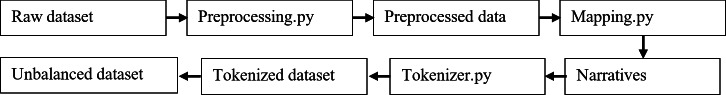
Data transformation pipeline for the distilled Bidirectional Encoder Representations from Transformers (DistilBERT) model, illustrating the workflow used in the retrospective study of 30-day all-cause readmission prediction using 50,000 deidentified encounters from the 2019 New York State Emergency Department Database. Starting from the raw dataset, Preprocessing.py cleans and standardizes records, Mapping.py translates Current Procedural Terminology and *International Classification of Diseases, 10th Revision* codes into their textual descriptions to create encounter narratives, and Tokenizer.py applies the DistilBERT-base-uncased tokenizer to produce the tokenized dataset used for model fine-tuning. The evaluation set retains the original class imbalance, while training rebalancing occurs outside this pipeline.

As illustrated in Figure 2, this pipeline began by converting each patient’s record into a descriptive paragraph. The diagram shows how the raw, structured CPT and *ICD-10* codes were first mapped to their corresponding textual meanings, official long-form descriptions for CPT codes and broader clinical chapter titles for *ICD-10* codes. This text was then combined with the numerical CCI score to form a unified text-based representation of each patient’s encounter. The final stage of the pipeline involved processing this narrative with the DistilBERT base uncased tokenizer, which lower-cases text by design; we inserted simple separators between code descriptions and the CCI so the sequence reflected the clinical order, truncated sequences to 512 tokens, and used dynamic per-batch padding so each batch was padded only to its longest example. This entire process was applied to both the limited and rich feature sets to create the 2 distinct text-based datasets required for the model. The pretrained DistilBERT-base-uncased model was then fine-tuned for 10 epochs using Adam with Decoupled Weight Decay (learning rate 2e-5, weight decay 0.01) with a per-device batch size of 4 for training and evaluation, evaluating and checkpointing at the end of each epoch and loading the best checkpoint based on validation *F*_1_-score. All hyperparameters were selected based on preliminary tuning experiments. All models were implemented in Python 3.10 using pandas, NumPy, scikit-learn, CatBoost, PyTorch, and the Hugging Face Transformers and Datasets libraries.

All 8 trained models (4 architectures times 2 feature sets) were evaluated on the untouched test set. Area under the receiver operating characteristic curve (AUROC) was used as the primary metric for both model selection during validation and final performance reporting. Pairwise comparisons of AUROC were conducted using DeLong test. *F*_1_-score and accuracy were also computed on the test set. Matthews correlation coefficient (MCC) was included as a balanced measure of classification performance that accounts for all 4 components of the confusion matrix. For each encounter, the model’s output was converted to a predicted probability of readmission.

### Sensitivity Analysis

We conducted a sensitivity analysis to evaluate how predictive performance changes as a function of the number of *ICD-10* and CPT codes. For this purpose, we constructed multiple feature configurations using the first k *ICD-10* and k CPT codes for k=1,2,3,4,5, in addition to the full code configuration including all available codes. For each configuration, the same train-test split was used to ensure paired comparisons across models. Also, for each feature configuration, a random forest classifier with fixed hyperparameters was retrained on the balanced training set and evaluated on the untouched test set. Model performance was assessed using AUROC as the primary metric, along with accuracy, precision, recall, *F*_1_-score, and MCC. Such an analysis was designed to assess whether predictive signals are concentrated in early code positions or distributed across the full set of structured codes.

### Ethical Considerations

This study used secondary data obtained from the HCUP, sponsored by the Agency for Healthcare Research and Quality. The dataset consists of administrative records derived from hospital billing and discharge data and does not contain direct personal identifiers. Because the data are deidentified and publicly available for research use under HCUP data use agreements, this study does not involve human subjects as defined by federal regulations and does not require institutional review board approval. This determination is consistent with guidance from the US Department of Health and Human Services for research involving deidentified data. No attempt was made to reidentify individuals, and all analyses were conducted in accordance with HCUP data use policies to ensure confidentiality and data security. No participant compensation was applicable for this study.

Generative artificial intelligence tools were used solely for language editing and improvement of grammar and clarity. All study design, analysis, results, and scientific content were developed and verified by the authors.

## Results

The analytic dataset consists of 50,000 encounters with a 30-day all-cause readmission rate of 21.1% (10,565/50,000). The mean patient age was 40.4 (SD 23.4) years, and 55.1% (27,570/50,000) of encounters involved female patients. The population is characterized by a low comorbidity burden, with a mean CCI of 0.35 (SD 0.84); 70% (38,518/50,000) of encounters had a CCI of 0, and 0.8% (392/50,000) had a CCI of 5 or greater. The distribution of coding complexity was similarly limited, with a mean of 7.65 (SD 7.68) CPT codes per encounter and 4.24 (3.35) *ICD-10* codes per encounter; 53.4% (26,716/50,000) of encounters having 5 or fewer CPT codes and 74.1% (37,064/50,000) having 5 or fewer *ICD-10* codes. The comprehensive performance metrics for all 8 experimental configurations, evaluated against a held-out test set of 10,000 patient encounters constructed to preserve the real-world 21.1% readmission prevalence, are presented below. These descriptive statistics are summarized in Table S1 in Multimedia Appendix 1.

For 3 of the 4 tested architectures, random forest, CatBoost, and the MLP, the models trained on the “limited feature set” achieved AUROC values comparable to or slightly higher than those trained on the “rich feature set.” As shown in Table 1, the random forest model, for instance, achieved a higher discriminatory power with limited data AUROC of 0.5596 (95% CI 0.5440‐0.5739) than it did with the full dataset AUROC of 0.5541 (95% CI 0.5393‐0.5671). Similarly, the MLP model showed a more pronounced advantage when using the limited data AUROC of 0.5386 (95% CI 0.5232‐0.5528) compared to the rich data AUROC of 0.5287 (95% CI 0.5156‐0.5430). DistilBERT was the only architecture where the rich feature set yielded a higher AUROC, at 0.5703 (95% CI 0.5565‐0.5842) compared to 0.5542 (95% CI 0.5411‐0.5666) on limited, though the CIs partially overlap.

**Table 1.** Performance metrics of all-cause 30-day all-cause readmission prediction models.

Model	Feature set	AUROC^a^ (95% CI)	*F*_1_-score (95% CI)	Accuracy (95% CI)	MCC^b^ (95% CI)
Random forest	Limited	0.5596 (0.5440‐0.5739)	0.2657 (0.2469‐0.2829)	0.7054 (0.6964‐0.7137)	0.0823 (0.0610‐0.1024)
Random forest	Rich	0.5541 (0.5393‐0.5671)	0.2528 (0.2351‐0.2699)	0.7110 (0.7019‐0.7192)	0.0761 (0.0536‐0.0975)
CatBoost	Limited	0.5369 (0.5236‐0.5518)	0.2555 (0.2381‐0.2723)	0.6795 (0.6698‐0.6888)	0.0514 (0.0312‐0.0713)
CatBoost	Rich	0.5333 (0.5191‐0.5470)	0.2509 (0.2343‐0.2682)	0.6752 (0.6665‐0.6842)	0.0438 (0.0247‐0.0633)
MLP^c^	Limited	0.5386 (0.5232‐0.5528)	0.2860 (0.2695‐0.3023)	0.6550 (0.6450‐0.6643)	0.0641 (0.0422‐0.0846)
MLP	Rich	0.5287 (0.5156‐0.5430)	0.2205 (0.2032‐0.2377)	0.7116 (0.7034‐0.7205)	0.0487 (0.0283‐0.0705)
DistilBERT^d^	Limited	0.5542 (0.5411‐0.5666)	0.3434 (0.3303‐0.3570)	0.4408 (0.4312‐0.4515)	0.0663 (0.0472‐0.0856)
DistilBERT	Rich	0.5703 (0.5565‐0.5842)	0.3406 (0.3275‐0.3543)	0.4893 (0.4796‐0.4987)	0.0723 (0.0530‐0.0910)

a AUROC: area under the receiver operating characteristic curve.

b MCC: Matthews correlation coefficient.

c MLP: multilayer perceptron.

d DistilBERT: distilled Bidirectional Encoder Representations from Transformers.

DistilBERT model trained on the rich dataset and the random forest model trained on the limited set achieved the highest AUROC scores. The DistilBERT model trained on the limited feature set yielded the highest *F*_1_-score of 0.3434 (95% CI 0.3303‐0.3570), driven by its high recall (0.7070). MCC values ranged from 0.0438 to 0.0823 across all configurations, reflecting the inherent difficulty of predicting all-cause readmission in a general population. The random forest model on limited data achieved the highest MCC (0.0823, 95% CI 0.0610‐0.1024).

Formal comparison of AUROC using DeLong test showed that differences between limited and rich feature sets were small in absolute magnitude (0.0036-0.0161).

Table 1 shows the performance metrics with 95% bootstrap CIs (1000 iterations) for 30-day all-cause readmission prediction models evaluated on a held-out test set of 10,000 deidentified emergency department encounters from the 2019 SEDD. Four ML architectures were each trained on limited (first 5 CPT and 5 *ICD-10* codes plus CCI) and rich (all available codes plus CCI) feature sets.

In addition, sensitivity analysis examining AUROC as a function of the number of retained *ICD-10* and CPT code positions showed that performance increased from 1 to 3 code positions, peaking at 0.5593 for the model using the first 3 *International Classification of Diseases* and 3 CPT codes, and then declined slightly for 4 codes (0.5558), 5 codes (0.5513), and the full code set (0.5460), see Figure 1 and Table S3 in Multimedia Appendix 1. MCC also followed a similar pattern, increasing from 0.0550 with 1 retained code position to 0.0774 with 3 retained positions. This pattern indicates a nonmonotonic relationship between the number of retained code positions and model discrimination. The absolute differences in AUROC across code counts were small (on the order of ~0.01‐0.02).

Figure 1 shows AUROC as a function of retained *ICD-10* and CPT code positions. Models were trained using the first k *ICD-10* and k CPT codes (k=1,2,3,4,5) with a full-code configuration including all available codes, with CCI included in all settings. Performance was evaluated on an untouched test set of 10,000 encounters with 21.1% readmission prevalence. AUROC increased from 0.5405 at k=1 to a peak of 0.5593 at k=3, then declined to 0.5558 (k=4), 0.5513 (k=5), and 0.5460 (full). Error bars represent 95% CIs estimated via bootstrap resampling (1000 iterations). These results indicate that predictive signal is concentrated in the first few code positions, with limited incremental benefit from additional codes.

Pairwise DeLong testing indicated a statistically significant difference between the 1-code and 3-code models (ΔAUROC≈0.0188; *P*=.02), whereas the difference between the 3-code model and the full-code model was not statistically significant (ΔAUROC≈0.0133; *P*=.06). This pattern is consistent with the primary comparison between limited and rich feature sets, where additional coding information did not yield consistent gains in discrimination.

## Discussion

### Principal Findings

The primary aim of this study was to test whether a limited set of structured clinical codes contains a clinically meaningful predictive signal for 30-day all-cause readmission. The results indicate that for 3 of 4 architectures (random forest, CatBoost, and MLP), models trained on the limited feature set achieved AUROC values comparable to their rich counterparts, with broadly overlapping 95% CIs. The random forest model achieved an AUROC of 0.5596 (95% CI 0.5440‐0.5739) on limited data versus 0.5541 (95% CI 0.5393‐0.5671) on rich data. DistilBERT was the only architecture where the rich feature set yielded a meaningfully higher AUROC (0.5703 vs 0.5542). The highest *F*_1_-score (0.3434) was achieved by DistilBERT on the limited feature set, driven by high recall (0.7070).

The observation that limited-feature models performed comparably to rich-feature models across most architectures suggests that the vast amount of additional data collected later in a hospital stay may act as statistical “noise” rather than a clear “signal” for this specific predictive task. Sensitivity analysis suggested that predictive discrimination was largely captured within the first few retained code positions, with AUROC peaking at 3 code positions and declining thereafter. This insight carries a profound strategic directive for hospital administration. In this study, adding more retained code positions did not produce consistent improvement in discrimination and, in the sensitivity analysis, performance was highest at 3 retained positions.

From an administrative and managerial perspective, the true value of this research lies not in the academic novelty of the models, but in the operational strategy they enable. The output of this work is not a simple binary classification of a patient as “high” or “low” risk, but rather a continuous, granular probability score generated for each patient encounter. This capability is a significant advancement over static, categorical risk tools like the LACE index, which are known to suffer from modest discriminatory power and often fail to identify a large fraction of patients who are ultimately readmitted [11]. A continuous probability score, however, serves as the engine for a modern, intelligent workflow, allowing hospital leadership to shift from a reactive, one-size-fits-all approach to a proactive, data-driven, and resource-optimized strategy. This enables a tiered approach to intervention, allowing for the precise and efficient allocation of finite clinical resources, as outlined in the framework below.

This tiered framework provides a clear and actionable value proposition for health care organizations (Table S2 in Multimedia Appendix 1). It directly addresses the dual mandate of improving patient outcomes while controlling costs, a central pressure point for modern hospital administration. By preventing avoidable readmissions, hospitals can mitigate significant financial penalties under value-based payment models like the HRRP, which have cost institutions billions of dollars since their inception [21].

It is essential to contextualize the performance of the developed models within the broader scientific landscape. In the absence of a direct internal comparison, our findings can be contextualized relative to published performance of the LACE index, which typically achieves c-statistics in the range of approximately 0.63-0.68 in general inpatient populations. The AUROC values observed in this study (approximately 0.53‐0.57) are lower than these reported LACE benchmarks. This difference is expected, as the present models rely exclusively on structured administrative coding data and do not incorporate additional variables included in LACE, such as prior use and admission characteristics. These values are also modest when compared to studies reporting AUROCs in the 0.70-0.85 range. This performance disparity, however, is not a weakness of the models but rather an expected and informative consequence of the study’s innovative design. First, predicting all-cause readmissions in a general, heterogeneous, and relatively healthy patient population, where the summary statistics between readmitted and nonreadmitted groups were nearly identical, is an inherently more difficult task than predicting condition-specific readmissions in a well-defined, high-risk cohort. Second, the models were intentionally constrained to a limited set of structured codes, rather than incorporating all available coding information. Finally, this study relied exclusively on structured billing codes, deliberately excluding the rich, contextual information embedded in unstructured clinical notes, which are known to significantly boost performance in models like ClinicalBERT [18].

Therefore, this study’s contribution is not a new state-of-the-art model in terms of raw accuracy, but rather the validation of an entirely different category of predictive tool: one designed for efficient risk stratification using restricted structured data.

In this context, the *F*_1_-score may be a more relevant metric of practical utility. The finding that the DistilBERT model trained on the limited feature set yielded the highest *F*_1_-score of 0.3434 suggests that it may offer the best balance between identifying at-risk patients and avoiding excessive false alarms. This configuration achieved a recall of 0.7070, meaning it could potentially flag approximately 70% of patients who will be readmitted, though at the cost of lower precision of 0.2267 (positive predictive value). In practice, health systems may need to calibrate the operating threshold based on their capacity for follow-up interventions and tolerance for false positives [22].

### Limitations

This study has several limitations that should be considered when interpreting the findings. First, the models were developed and tested using data from a single state (New York) and a single year (2019). The performance of predictive models can vary across different populations, health care systems, and time periods. External validation on geographically and demographically distinct cohorts is necessary before any consideration of broader deployment.

A key limitation of this study is the reliance on eligibility criteria based on key variables required to define encounters and outcomes. Eligible encounters were defined using valid VisitLink identifiers and nonmissing DaysToEvent values to ensure a well-defined outcome and consistent encounter structure. However, in real-world deployment, predictive models must operate in the presence of incomplete and evolving data. The current framework does not fully capture the challenges of prediction under incomplete data conditions. Future work should evaluate model robustness to missing inputs and consider approaches such as imputation or models designed to handle partially observed data without requiring prior filtering.

Another limitation is the use of data from 2019. Although the structure of administrative coding systems such as *ICD-10* and CPT remains stable, EHR systems, documentation practices, and hospital workflows have continued to evolve since 2019. These changes may affect data availability and completeness, which may influence model performance in more recent settings. Therefore, external validation using more recent datasets is necessary to assess the continued applicability of the findings. Although this approach ensures a well-defined outcome, it may differ from real-world deployment settings where models must operate on partially incomplete data streams without prior eligibility filtering. Periodic recalibration would be necessary if these models were deployed in current clinical environments.

A key limitation is that the dataset did not include timestamps for individual diagnosis or procedure codes. Therefore, although the limited feature set was defined using earlier code positions, we cannot confirm that these codes were available at the beginning of the encounter or that the models constitute true early prediction. The results should therefore be interpreted as comparing restricted versus more complete structured coding representations collected during the hospital visit, rather than temporally validated early versus later prediction models. While this controlled design enables a clear comparison between restricted and more complete representations, it may not fully reflect the complexity of real-world data generation processes. Future research could extend this work by developing alternative feature construction strategies that incorporate additional information.

Readmissions occurring outside the data coverage area, such as in other states or nonparticipating facilities, are not captured in the dataset and are treated as nonreadmissions. This may lead to underestimation of the true readmission rate and could bias model performance estimates, particularly by misclassifying some positive cases as negative. Because this limitation applies consistently across all models, it does not affect the relative comparison between limited and rich feature settings, but it may influence absolute performance estimates.

Model evaluation in this study focused primarily on discrimination metrics and did not include formal assessment of calibration or clinical utility. While AUROC and *F*_1_-score provide information on the model’s ability to distinguish between readmitted and nonreadmitted patients, they do not assess whether predicted probabilities are well aligned with observed outcomes or whether the model would improve clinical decision-making in practice. Future work should include calibration analysis, such as calibration plots or summary measures, and evaluation of clinical utility using decision-curve analysis or net-benefit approaches to determine whether these models provide meaningful advantages over simple rules or existing tools. This study did not include a formal algorithmic fairness audit or subgroup analysis across demographic categories such as age, sex, race/ethnicity, or insurance status, and it must be stated unequivocally that such an audit is an absolutely necessary prerequisite before any consideration of clinical deployment. Historical administrative data can embed and perpetuate patterns of systemic bias in health care access and treatment [23]. A model trained on such data risks learning these biases and systematically underestimating the readmission risk for marginalized racial, ethnic, or socioeconomic groups, a prominent risk given that safety-net hospitals serving these populations are already disproportionately penalized by the HRRP [24]. Deploying an unaudited algorithm is, therefore, not just an ethical failure; it is a profound operational and financial risk. Such a tool could lead to the systematic denial of supportive resources to the very populations who need them most, worsening health inequities while simultaneously failing to reduce the readmission rates that drive financial penalties. A rigorous fairness audit, comparing key metrics like false positive and false negative rates across protected demographic subgroups, is a fundamental risk mitigation strategy required to ensure that the model achieves its intended clinical and financial goals equitably.

Because multiple encounters from the same VisitLink were included as separate observations, the analysis may contain dependence among observations from the same patient. No adjustment for this dependence, such as patient-level clustering, was applied in the current analysis.

### Future Directions

A key direction of future research is to enrich the model with more diverse data signals. The next logical step is to integrate unstructured clinical notes using natural language processing, leveraging the clinical nuance and detailed patient narratives that are absent in billing codes and have been shown to significantly improve readmission prediction [18]. Even more critical is the incorporation of data on social determinants of health. A growing body of evidence confirms that factors like housing instability, transportation access, and food insecurity are powerful predictors of readmission [25]. This research direction is not only scientifically promising but also aligns directly with evolving health care policy. CMS is actively expanding social determinants of health data collection requirements, and the 2025 Inpatient Prospective Payment System Final Rule elevates housing instability to a complication or comorbidity status, directly impacting reimbursement to the hospital [26]. Aligning future model development with these regulatory trends is essential for creating tools that are both effective and relevant to the administrative realities of modern health care.

The next steps toward clinical translation include external validation on diverse cohorts, formal calibration and fairness audits, integration of additional data sources such as clinical notes and social determinants of health [25,26]. Our findings are consistent with recent work by Lin et al [27], who demonstrated that a random forest model built from structured EHR data achieved marginally better identification of high-risk readmissions compared to standardized risk tools. A recent systematic review by Selmer et al [28] further confirmed that readmission prediction models commonly incorporate demographic variables, comorbidities, and prior hospitalizations, while noting that social determinants of health remain inconsistently evaluated, aligning with the future directions identified here. Ultimately, the gold standard for clinical translation remains a prospective, cluster-randomized controlled trial to measure impact on readmission rates, costs, and clinician satisfaction [29].

The path from retrospective proof to prospective impact requires further validation. The gold standard for determining true clinical and administrative use can only be achieved through a prospective, cluster-randomized controlled trial. Such a study, deploying the risk stratification tool in some hospital units but not others, would definitively measure its real-world impact on key outcomes such as 30-day all-cause readmission rates, total cost of care, and clinician satisfaction, providing the final and most important piece of evidence for its value in transforming hospital operations [29].

### Conclusion

This study provides evidence that a clinically meaningful predictive signal for 30-day all-cause readmissions is present within a limited set of structured clinical codes, with performance to models using more comprehensive coding information.

For hospital leadership, this research may support a strategic shift from reactive post-discharge analysis toward proactive, data-driven resource allocation during the patient’s stay. By generating a risk score for each patient encounter, health systems can implement a tiered intervention framework, deploying clinical resources based on the predicted risk. This approach suggests a pathway to simultaneously improve care quality, enhance operational throughput, and mitigate the substantial financial penalties associated with preventable readmissions.

## Supplementary material

10.2196/83918 Multimedia Appendix 1 Supplementary tables and figures.

## Abbreviations

AUROC: area under the receiver operating characteristic curve
CCI: Charlson Comorbidity Index
CMS: Centers for Medicare & Medicaid Services
CPT: Current Procedural Terminology
DistilBERT: distilled Bidirectional Encoder Representations from Transformers
EHR: electronic health record
HCUP: Healthcare Cost and Utilization Project
HRRP: Hospital Readmissions Reduction Program
*ICD-10*: *International Classification of Diseases, 10th Revision*
LACE: length of stay, acuity of the admission, comorbidity of the patient, and emergency department use
MCC: Matthews correlation coefficient
ML: machine learning
MLP: multilayer perceptron
SEDD: New York State Emergency Department Database
