# Supplementary material for: Prediction of 30-Day All-Cause Hospital Readmissions Using Limited Structured Electronic Health Record Data: Retrospective Comparative Study
Source: JMIR Form Res. 2026 May 22;10:e83918. doi: 10.2196/83918 (PMC13197155; doi:10.2196/83918)
Supplement: Multimedia Appendix 1 [file formative-v10-e83918-s001.docx]

# **Appendix**

| Table S1: Comparative Analysis of Readmitted vs. Non-Readmitted Groups. | | |
| --- | --- | --- |
| Characteristic | Readmitted Group (N=10,565) | Non-Readmitted Group (N=39,435) |
| Mean CCI | 0.40 | 0.35 |
| Mean # of CPT Codes per Encounter | 7.71 | 7.64 |
| Mean # of ICD-10 Codes per Encounter | 4.36 | 4.20 |

*Table A1 shows the comparative analysis of readmitted versus non-readmitted groups among 50,000 de-identified emergency department encounters from the 2019 New York SEDD. Readmitted group (n=10,565): Mean CCI 0.40, Mean CPT codes 7.71, Mean ICD-10 codes 4.36. Non-readmitted group (n=39,435): Mean CCI 0.35, Mean CPT codes 7.64, Mean ICD-10 codes 4.20. The near-identical summary statistics between groups demonstrate that simple statistical measures are insufficient to distinguish high-risk from low-risk patients.* *CCI, Charlson Comorbidity Index; CPT, Current Procedural Terminology; ICD-10, International Classification of Diseases, 10th Revision.*

| Table S2: A Proposed Framework for Risk-Stratified Resource Allocation. | | | | |
| --- | --- | --- | --- | --- |
| Risk Tier | Predicted Probability | Triggered Action/Intervention | Resource Intensity | Strategic Goal |
| Tier 1:  Low Risk | < 15% | Standard discharge protocols. | Low | Prevent over-utilization of resources on patients unlikely to benefit. |
| Tier 2: Moderate Risk | 15% - 30% | Automated, low-cost interventions (e.g., enrollment in post-discharge call queue, tailored SMS/portal messages). | Low to Medium | Scalable, efficient support to a broad group with marginal risk. |
| Tier 3: High Risk | 30% - 50% | Automated flag on a clinical dashboard, prompting targeted review by a case manager or discharge planner. | Medium to High | Focus skilled personnel on ensuring robust discharge plans for at-risk patients. |
| Tier 4: Very High Risk | > 50% | Immediate, active in-hospital consultation (e.g., clinical pharmacist, social worker, care transition coordinator). | High | Reserve most intensive and costly resources for the highest-risk patients. |

Table A2 shows the proposed framework for risk-stratified resource allocation based on predicted 30-day readmission probability from machine learning models trained on minimal early-encounter data (first 5 ICD-10 and 5 CPT codes plus CCI) from 50,000 de-identified emergency department encounters in the 2019 New York SEDD. Risk tiers are illustrative and would require calibration to local clinical workflows and resource availability.


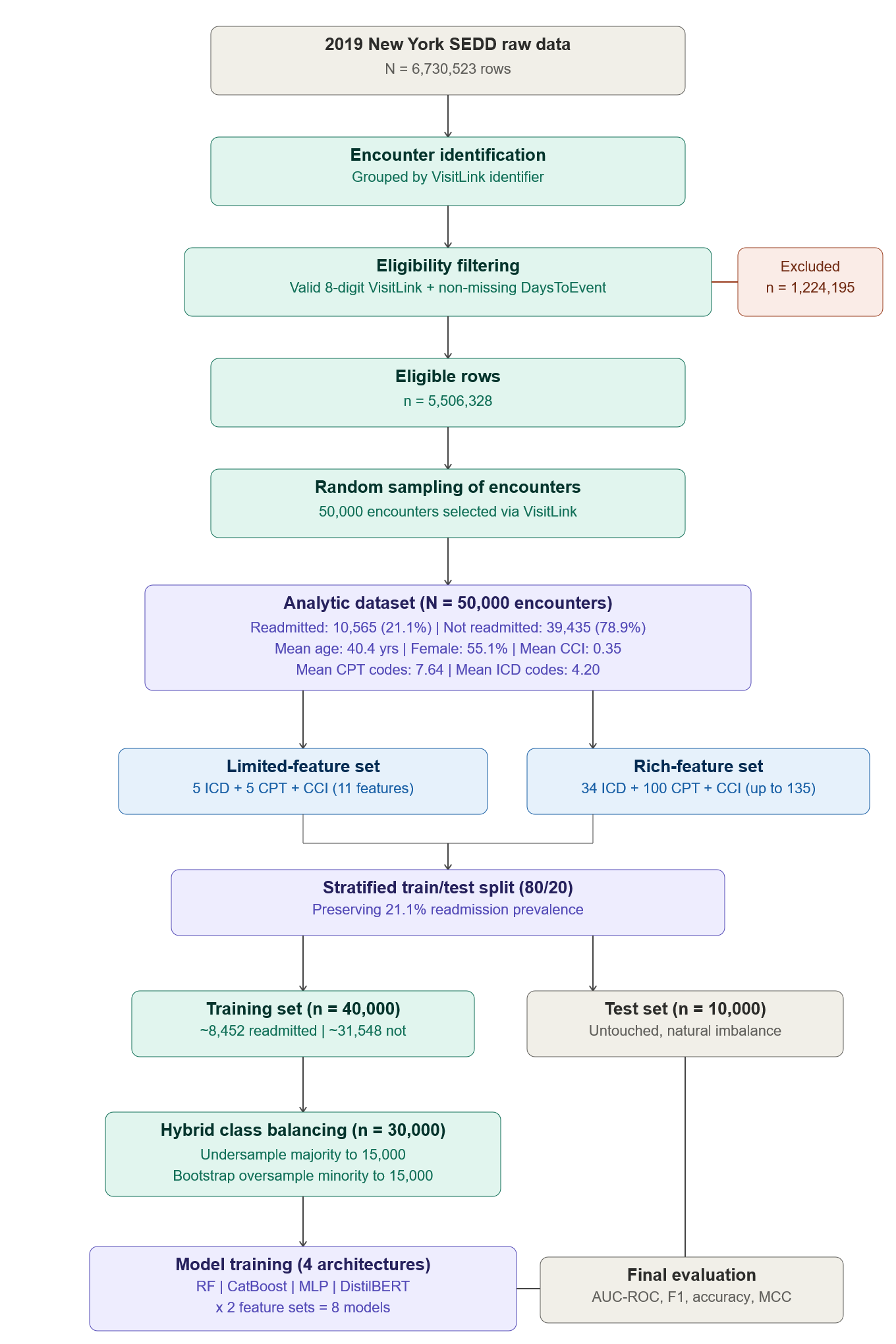


Figure A1: The flowchart of construction pipeline.

*Figure S1 follows a CONSORT-style format to improve transparency of the cohort construction process. SEDD: State Emergency Department Database; ICD-10: International Classification of Diseases, 10^th^ Revision; CPT: Current Procedural Terminology; CCI; RF: Random Forest; MLP: Multi-Layer Perceptron; AUC-ROC: Area Under the Receiver Operating Characteristic Curve; MCC: Matthews Correlation Coefficient.*

| 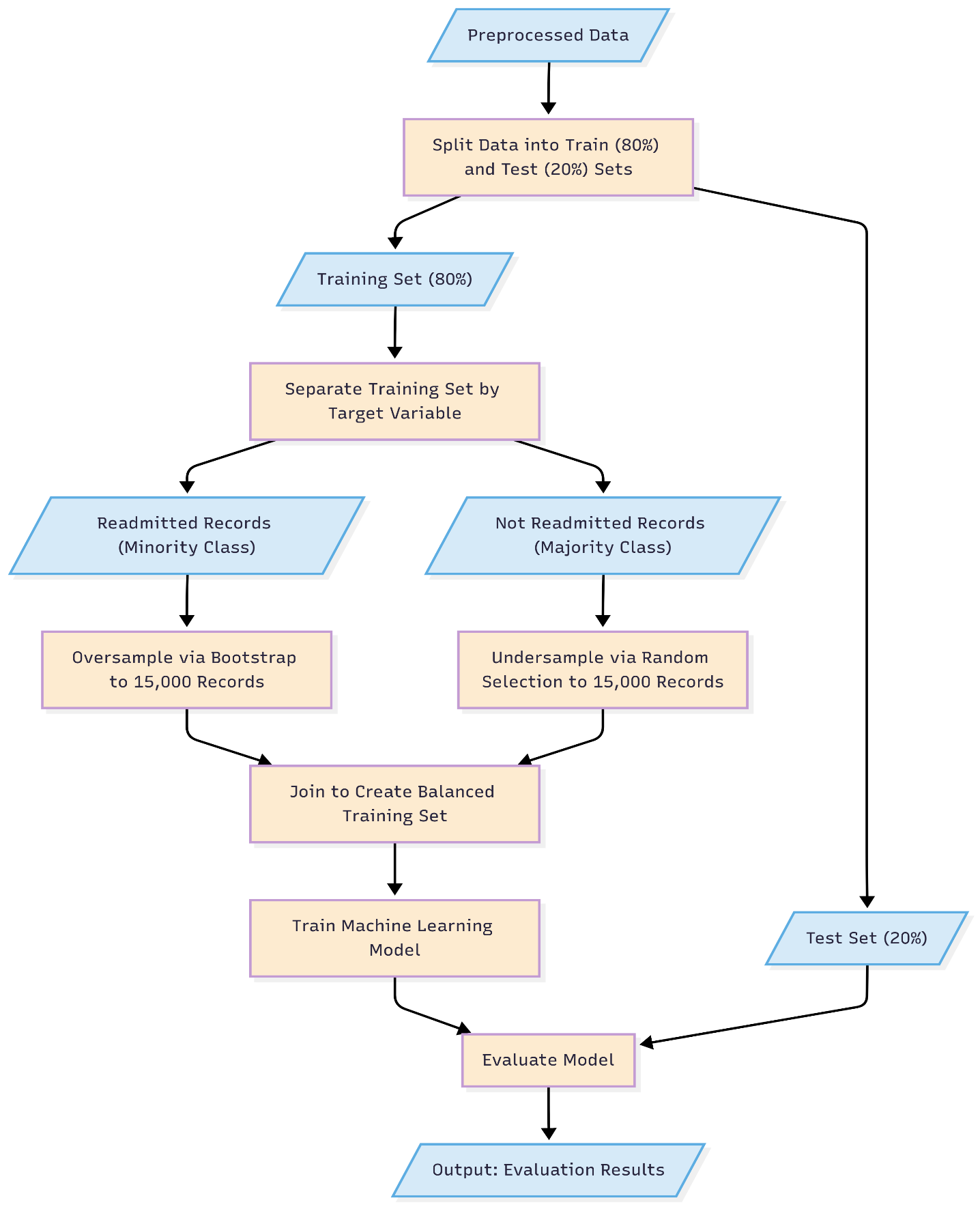 |
| --- |
| Figure A2: Overall experimental workflow. |

Figure S2 shows the overall experimental workflow for the retrospective comparative study of 30-day all-cause hospital readmission prediction using 50,000 de-identified emergency department encounters from the 2019 New York State Emergency Department Database (SEDD).

| Table S3: Performance Metrics Across Retained ICD-10 and CPT Code Positions. | | | | | | | |
| --- | --- | --- | --- | --- | --- | --- | --- |
| Feature setting | AUROC | 95% CI | Accuracy | Precision | Recall | F1 | MCC |
| Codes 1 to 1 | 0.5405 | 0.5262–0.5553 | 0.5887 | 0.2397 | 0.4359 | 0.3093 | 0.0550 |
| Codes 1 to 2 | 0.5544 | 0.5407–0.5686 | 0.6454 | 0.2545 | 0.3516 | 0.2953 | 0.0680 |
| Codes 1 to 3 | 0.5593 | 0.5446–0.5742 | 0.6788 | 0.2680 | 0.3005 | 0.2834 | 0.0774 |
| Codes 1 to 4 | 0.5558 | 0.5417–0.5698 | 0.6925 | 0.2699 | 0.2669 | 0.2684 | 0.0737 |
| Codes 1 to 5 | 0.5513 | 0.5366–0.5657 | 0.7011 | 0.2702 | 0.2437 | 0.2563 | 0.0700 |
| Full | 0.5460 | 0.5323–0.5597 | 0.6765 | 0.2641 | 0.2972 | 0.2797 | 0.0722 |

*Table A3 shows the performance of a Random Forest model evaluated across feature configurations using the first k ICD-10 and k CPT code positions (k = 1,2,3,4,5) and a full-code configuration including all available codes. Models were trained on a balanced training set and evaluated on an untouched test set of 10,000 encounters with 21.1% readmission prevalence. AUROC is reported with 95% confidence intervals estimated via bootstrap resampling (1,000 iterations). Additional metrics include accuracy, precision, recall, F1 score, and MCC. Results show that performance peaks at k = 3 and declines slightly as additional code positions are included.*
